# Seroprevalence and Associated Risk Factors of Bovine Brucellosis in District Gujranwala, Punjab, Pakistan

**DOI:** 10.3390/ani11061744

**Published:** 2021-06-11

**Authors:** Muhammad Rashid Khan, Abdul Rehman, Salman Khalid, Mansur Ud Din Ahmad, Muhammad Avais, Mobeen Sarwar, Farhat Nazir Awan, Falk Melzer, Heinrich Neubauer, Tariq Jamil

**Affiliations:** 1 Department of Epidemiology and Public Health, University of Veterinary and Animal Sciences, Lahore 54000, Pakistan; rashidgadi1327@gmail.com (M.R.K.); mansuruddin@uvas.edu.pk (M.U.D.A.); 2 Directorate of Animal Disease Diagnostics, Reporting and Surveillance, Livestock and Dairy Development Department Punjab, Lahore 54100, Pakistan; salmankhalidonline@gmail.com (S.K.); mobeensarwarpne@gmail.com (M.S.); dadrs786@gmail.com (F.N.A.); 3 Department of Veterinary Medicine, Faculty of Veterinary Science, University of Veterinary and Animal Sciences, Lahore 54000, Pakistan; mavais@uvas.edu.pk; 4 Institute of Bacterial Infections and Zoonoses, Friedrich-Loeffler-Institut, 07743 Jena, Germany; falk.melzer@fli.de (F.M.); heinrich.neubauer@fli.de (H.N.); tariq.jamil@fli.de (T.J.)

**Keywords:** brucellosis, zoonosis, seroprevalence, bovines, Pakistan

## Abstract

**Simple Summary:**

Our study estimated the seroprevalence of brucellosis in bovine herds and identified the important risk factors associated with the occurrence of the disease at livestock farms. A total of 220 sera from 46 bovine herds of district Gujranwala, Punjab, Pakistan, was collected and subjected to brucellosis screening by serology. It resulted in 58.7% herd-based and 22.7% individual animal-based seroprevalence. Age, herd size and previous history of abortion came out as associated risk factors. Strict biosecurity, personal protection, quarantine measures and routine screening are recommended at livestock-holdings/farms, whereas disease awareness and pasteurization of milk are recommended for the general population to prevent zoonosis.

**Abstract:**

Bovine brucellosis is a contagious zoonotic disease that causes economic losses through abortion and infertility. A cross-sectional study was designed to determine the seroprevalence and associated risk factors of bovine brucellosis in district Gujranwala of Punjab, Pakistan. A total of 220 bovine sera (112 from buffaloes, 108 from cattle) from 46 unvaccinated herds were collected. Parallel testing by the Rose Bengal Plate Test (RBPT) and Indirect Enzyme-linked Immunosorbent Assay (I-ELISA) showed a 58.7% (27/46) herd-level and 22.7% (50/220) animal-level seroprevalence. Seroprevalence was higher (*p* < 0.001, OR = 7.62) in adult animals (37.2%) compared to younger animals (4.9%). A herd size of >10 animals (*p* = 0.021, OR = 7.83), less housing space (*p* = 0.037, OR = 6.39) and history of abortion at the farm (*p* = 0.023, OR = 5.6) were found as risk factors associated with the seropositivity of brucellosis. There was a substantial agreement between the RBPT and I-ELISA results (Cohen’s kappa coefficient (κ) = 64.16, percent agreement = 89.5%). In conclusion, a relatively higher seroprevalence was found compared to the previous reports from the country. Standardization and validation of the advanced diagnostic tests would be needed. Biosecurity, personal protection, quarantine measures and routine screening of animals at the farm level and disease awareness programs and consumption of pasteurized milk in the human population will be helpful in preventing the transmission/zoonosis of the disease.

## 1. Introduction

Brucellosis is an infectious zoonotic disease caused by the bacteria of the genus *Brucella*. These are Gram-negative, non-capsulated, non-motile and facultative intracellular coccobacilli that mainly infect cattle, buffaloes, goats, sheep and pigs worldwide [1]. These bacteria have host preferences, e.g., *Brucella (B.) abortus* prefers bovines, *B. melitensis* sheep and goats, *B. suis* pigs, *B. ovis* rams and *B. canis* dogs [2,3]. Among them, *B. abortus*, *B. melitensis*, *B. suis* and *B. canis* can infect non-preferred hosts and humans [1,4,5]. It is one of the most frequently transmitted zoonosis in the world [6]. According to the World Animal Health Information Database maintained by the World Organization for Animal Health (OIE), the disease is prevalent in domestic animals in various parts of the world, e.g., Asia, Africa and Latin America, while several countries have successfully eradicated it through vaccination, screening and culling programs, at least in domestic animals [7,8].

Bovine brucellosis is highly contagious and is transmitted via direct contact with infected animals or indirectly via contaminated fomites. Humans usually get infection via contact with abortive (and birth) fluids, mucous membranes (e.g., mouth, eyes) and consumption of contaminated raw milk or milk products from infected animals [9,10]. This disease is characterized by abortion in the last trimester followed by retention of fetal membranes in animals. It can stay asymptomatic and may remain undiagnosed. In humans, it is mostly a chronic malaise [11]. However, in acute cases, it may cause undulant fever accompanied by occasional abortion and endometritis in women and orchitis and epididymitis in men [11]. Infected animals may remain carriers for their life and shed the bacteria when immunocompromised [2]. Several factors have been associated with the occurrence of bovine brucellosis at livestock farms [12,13]. These animal-level factors include species, sex, age, genetics, immunity and body condition score [14,15]. In turn, farm-level factors include breeding practices, animal replacement, hygiene practices, testing and culling of the seropositive animals, feeding and grazing practices and type of the farming system—whether a mixed or single animal species [16].

Livestock plays an important role in the economic survival of the rural population of Pakistan [17]. The dairy sector in Pakistan can be divided into three categories based on the herd size, i.e., small animal holders (less than 10 animals), medium-size animal holders (10–30 animals) and large-scale farmers (more than 30 animals), contributing 51%, 29%, and 20% to the national milk production, respectively [18,19]. Despite the economic and zoonotic significance of brucellosis, it has remained an underrated infection where farmers often ignore implementing the effective preventive and control measures at the livestock farms. Common diagnostic tests used in the country include the Rose Bengal Plate Test (RBPT), Enzyme-Linked Immunosorbent Assay (ELISA), Milk Ring Test (MRT) and Serum Agglutination Test (SAT) followed by Polymerase Chain Reaction (PCR) for species-level identification [20,21,22]. Isolation of Brucellae remains the gold standard but is hazardous, not very efficient and requires advanced technical expertise and biosafety levels (BSLs), e.g., BSL 3, which is not readily available in the country. There is no 100% safe treatment for animals and vaccination of brucellosis is scarcely practiced in farm animals in Pakistan.

Bovines (buffaloes and cattle) in Pakistan contributed significantly to the total livestock population (43.48%; 87.8/201.9 million heads) and to the total dairy milk production (96.8%; 59.7/61.7 million tons) in 2019 [23]. As the main zoonotic transmission route of brucellosis is by consumption of contaminated unpasteurized dairy milk, and >97% of the dairy milk in Pakistan is marketed as raw/unpasteurized, we were interested to know the situation and associated risk factors of bovine brucellosis in small and medium-sized herds of district Gujranwala, of Punjab, Pakistan. This study was expected to alert the concerned authorities as to their possible role in the control and eradication of bovine brucellosis and subsequently its zoonotic transmission in this district.

## 2. Materials and Methods

### 2.1. Study Population and Sampling

Based on the bovine population of the districts in the semi-arid agro-ecological zone of Pakistan, we selected district Gujranwala for having the highest bovine population (196,259 cattle and 575,503 buffaloes) among all the districts in that zone [24]. The district is located between 32.1877° N and 74.1945° E and is about 80 km to the north of the provincial capital, Lahore. It is 226 m (744 ft.) above sea level and has a hot and semi-arid climate. The district has 834 Mouzas (villages) where most farmers are small dairy holders. This study focused the small and medium-sized subsistence and semi-commercial animal holdings/farms, which make up more than 97% of the total livestock population in the country. There was no vaccination history against brucellosis in these animal holdings/farms.

**Figure 1:**
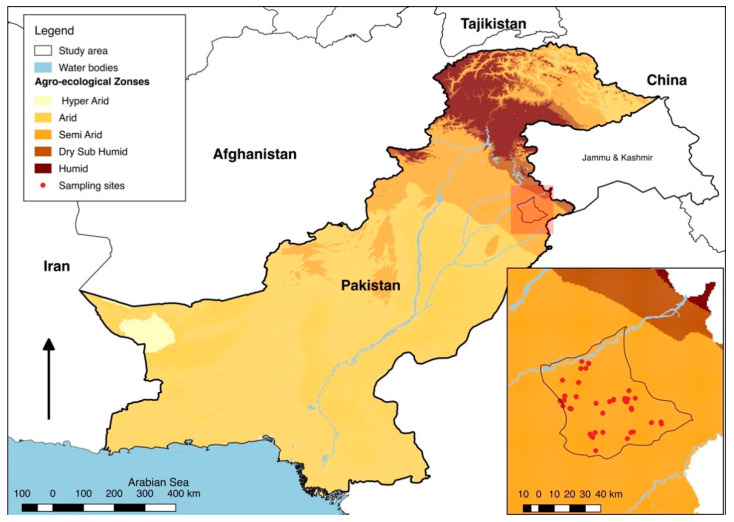
Map showing the agro-ecological zones of Pakistan based on aridity. The inset map shows the distribution of livestock farms in the study area (the map was produced by the corresponding author (AR) using QGIS).

A cross-sectional study was designed to estimate the seroprevalence of brucellosis and the potential risk factors associated with the seropositivity. The sample size was calculated for a single proportion considering a large population, assuming a 14.9% prevalence, as reported by Nasir et al. [25], with 95% confidence and 5% desired precision levels, resulting in at least 195 animals to be sampled (https://epitools.ausvet.com.au/oneproportion, accessed on 10 October 2018). Neither a declared list of the livestock farms nor animal identification data were available in the district; therefore, a convenience sampling technique for the selection of the farms was used (Figure 1). A genuine attempt was made to include farms from all different parts of the district. However, at each farm, the animals were selected at random. Consequently, a total of 220 blood samples (buffaloes = 112, cattle = 108) were collected from apparently healthy animals from 46 small (herd size ≤10 animals) and medium-sized (herd size >10 animals) farms during February and March 2019. Blood samples (~3 mL from each animal) were collected in blood collection tubes with clot activator (Atlas–Labovac Italiano, FL Medical, Torreglia PD, Italy). Subsequently, the serum was separated by centrifugation and stored at −20 °C [26]. Afterwards, the serum samples were transported to the Provincial Disease Diagnostic Laboratory, Directorate of Animal Disease Diagnostics, Reporting and Surveillance, Livestock and Dairy Development Department Punjab, Lahore, Pakistan, for further processing.

### 2.2. Rose Bengal Plate Test (RBPT)

The sera were initially screened by Rose Bengal-stained *Brucella* antigen (Strain-99) following the manufacturer’s instructions (Veterinary Research Institute (VRI), Lahore, Pakistan). Briefly, 30 μL of each serum sample was mixed with 30 μL of RBPT antigen on a transparent glass slide for 4 mins at room temperature. The reaction was assessed positive if agglutination occurred and negative if no agglutination was visible.

### 2.3. Indirect Enzyme-Linked Immunosorbent Assay (I-ELISA)

Additional to RBPT, the sera were subjected to parallel testing by ID Screen^®^ brucellosis serum indirect multi-species (IDVet, Grabels, France) ELISA kit with known sensitivity (100% (95% CI: 89.57–100%)) and specificity (99.74% (95% CI: 99.24–99.91%)) for the presence of IgG antibodies against *B. abortus*, *B. melitensis*, and *B. suis*, as recommended by the manufacturer [27]. The sera were considered negative when the % S/P < 110%, suspicious when 110% ≤ % S/P < 120%, and positive when % S/P ≥ 120%.

### 2.4. Epidemiological Data Acquisition and Statement of Ethics

A structured questionnaire was developed to collect the data related to the animals: sampling, husbandry practices on the farm, housing facilities and practices by animal handlers that could be associated with the transmission of brucellosis in humans or other animals. These questions were about (i) farm type, (ii) herd size, (iii) presence of other animal species on the farm, (iv) frequency of the visits of veterinary or para-veterinary staff, (v) presence of fencing, (vi) feeding practices, (vii) neighboring livestock farms, (viii) breeding practices, (ix) abortion history on the farm during the last six months, (x) handling of aborted animals and (xi) animal quarantine. The data were collected on hard proformas through personal interviews by the first author in the local language, i.e., Punjabi, with the help of field veterinary staff from the Directorate of Animal Disease Diagnostics, Reporting and Surveillance, Livestock and Dairy Development Department Punjab, Lahore, Pakistan. Before filling the questionnaire, the study was explained to the farmers and verbal consent to participate in the study was obtained. All the data were carefully entered into Microsoft Excel sheets and processed for statistical analysis.

### 2.5. Statistical Analysis

Statistical analyses were performed using R software version 4.0.4 for Mac and RStudio version 1.4.1106 as an interface [28,29]. Samples seropositive for any of the two tests (RBPT and I-ELISA) were considered positive for brucellosis (parallel testing approach). Animal-level prevalence was calculated by dividing the number of positive animals by the total number of animals screened. However, a herd was considered positive if it had at least one positive animal with any of the two tests. Effect of various explanatory variables—either farm-level or animal-level—on seroprevalence of brucellosis was assessed using multivariable logistic regression analysis. For this, we excluded all variables that were practiced the same way by all farms, e.g., “quarantine measure” was excluded as no farm had practiced quarantine. Herd size was treated as a categorical variable. All the potential explanatory variables were included with an additive mode in a multivariable model. Two separate multivariable models—one for the farm-level and the other for the animal-level variables—were built to avoid the inflation effect. The initial multivariable model for animal-level factors included four, whereas for the farm-level factors it included nine variables. The ultimate models were fitted with the response variable as positive or negative. Both models were run using the glm function and non-significant variables (*p* > 0.05) were removed one by one through a manual stepwise backward single-term deletion using drop1 function, starting with the highest *p*-value until the variables left had a *p* < 0.05. The link function “logit” was used to report (1) the coefficient; (2) the ratio of the coefficient to its standard error; and (3) the *p*-value. Odds ratios (OR) along with 95% confidence intervals (CI) for the final model were calculated using the exp function. Akaike information criterion (AIC) values were used to assess the quality of the model fit. Deviance residuals were also examined for homoscedasticity and a normal distribution. The final model for the animal-level factors contained one variable, while for the farm-level factors it contained three variables. The Cohen’s kappa coefficient with 95% CI and percentage of positives with congruent classifications were used to see the extent of the agreement in the two tests (RBPT and I-ELISA). A map showing the study area and sampling sites was produced using QGIS 2.16.3 (QGIS.org, 2021, QGIS Geographic Information System, QGIS Association). The updated base map for Gujranwala District, as previously described, was used [19].

## 3. Results

### 3.1. Study Population

A total of 220 bovines (112 buffaloes and 108 cattle) from 46 livestock holdings in district Gujranwala were screened for brucellosis by RBPT and I-ELISA. The median herd size of the selected livestock farms was 13 (Q1–Q3: 8–21), while the median number of animals included in the sampling from each farm was 5 (Q1–Q3: 3–6). The majority of the animals (85.9%) were female. The median age of the animals was 4 years ((Q1–Q3 = 3–6 years), (median age for buffaloes = 5 years and for cattle = 4 years)). The median age of the seropositive animals was 5 years (Q1–Q3 = 4–6), while for the seronegative it was 4 years (Q1–Q3 = 3–6).

### 3.2. Seroprevalence of Brucellosis

Parallel investigation showed 22.7% (50/220, 95% CI: 17.5–28.9%) animal- and 58.7% (27/46, 95% CI: 43.3–72.7%) herd-level seroprevalence (Table 1). Three samples positive by RBPT showed a negative reaction by I-ELISA and 20 samples vice versa (Supplementary Materials Tables S1–S3).

Cohen’s kappa coefficient showed a substantial agreement (percent agreement = 89.54; Cohen’s kappa coefficient = 0.64, SE = 0.067, 95% CI = 0.51–0.77) between these two tests [30,31]. However, there was a significant difference (χ2 = 7.619, *p* = 0.005) between RBPT and I-ELISA to detect seropositivity in these samples. In our study, the relative sensitivity and specificity of RBPT compared to I-ELISA was 57.45% (95% CI = 42.18–71.74%) and 98.27% (95% CI = 95.02–99.64%), respectively.

### 3.3. Risk Factors Associated with the Seroprevalence of Brucellosis

#### 3.3.1. Animal-Level Risk Factors

The multivariable analysis revealed that the seroprevalence was significantly higher (*p* < 0.001) in adults (27.1%) compared to the younger animals (4.7%) and the odds for being positive were seven times higher (OR = 7.63, 95% CI = 2.22–47.93) in adults than the younger ones. Of the 189 female animals tested, 48 were positive. Of the 31 males, only two tested positive (Table 2). The seropositivity was higher in females (25.4%) compared to males (6.5%); however, this association was not statistically significant. The AIC of the initial model was 231.1 while for the final model it was 227.0.

#### 3.3.2. Farm-Level Risk Factors

The descriptive analysis of the farm-level variables is presented in Table 3. Most of the farmers (63%) followed the traditional farming system (where animals are reared on agricultural by-products to fulfil household dairy demands), while a small number of farmers (37%) raised animals in a semi-intensive commercial-type farming system. Regarding breeding practices, artificial insemination was practiced mainly (84.7%) in cattle, while natural mating was the common breeding method (93.5%) in buffaloes. Most of the farmers (66.3%) used semen from private companies, whereas a small portion (20.7%) used government supply, while the remaining (13%) relied on natural mating. Most of the farmers (86.67%) did not follow precautionary measures when handling the aborted fetuses. About 55% of the farmers reported that they disposed of the aborted fetuses in the countryside and 40% reported burying the aborted fetus. One out of 20 farms where abortion occurred, reported that the fetus was thrown in a nearby water canal. Only one farmer reported disinfection of the site after abortion, while most of the farmers (95%) did not disinfect the contaminated area at all. Most of the farmers (75%) retained the aborting animals at their farms; however, 25% of the farmers sold out these animals in the market subsequently.

The multivariable analysis showed that the odds of seropositivity was seven times higher (*p* = 0.021, OR = 7.83, 95% CI = 1.48–52.43) on farms where the herd size was comparatively larger, i.e., >10 animals (Table 4). Similarly, the farms with the smaller area had six times higher seroprevalence as compared to farms with large housing space (*p* = 0.037, OR = 6.39, 95% CI = 1.21–43.26). Moreover, the history of abortion at the farm during the last six months was strongly associated with the seroprevalence of brucellosis at a livestock farm (*p* = 0.023, OR = 5.6, 95% CI = 1.29–31.29). The initial herd model was run with nine variables. The AIC value of the initial model was 66.24, while for the final model it was 55.26.

## 4. Discussion

Brucellosis is a zoonotic disease mainly of the reproductive system of bovines. The disease is considered endemic in Pakistan [32,33]. The situation in animals is a direct indication of zoonotic risk posed towards human health. RBPT remains one of the cheapest and readily available tests for screening brucellosis globally. I-ELISA can be used as a single screening test but would require standardization and validation. RBPT and I-ELISA detect common anti-smooth-lipopolysaccharide antibodies against *B. abortus* and *B. melitensis* and are often not able to differentiate between vaccinated and diseased animals [34,35]. However, species-specific detection and vaccinal/field strain differentiation can be achieved by PCR or culture. Both tests (RBPT and I-ELISA) can be used individually for brucellosis screening purposes; nevertheless, they may require complementary confirmation by secondary tests [35,36,37,38]. Hence, standardization and validation of the tests are necessary depending upon the disease situation and available resources to detect false negative/positive animals. We calculated Cohen’s kappa coefficient to compare the performance of both tests and found a substantial agreement. These results agreed with previous studies from Pakistan and neighboring countries [26,35,39,40].

At the animal level, the seroprevalence was 22.7%. A more recent study from the same region also reported similar results (27.86%) in crossbred cattle on three large dairy farms [41]. However, a wide range of seroprevalence (3.3–28.9%) in bovines was reported during the last five years by various studies in different regions and production systems [14,22,33,39,41,42]. Even a wider range has been reported in the neighboring countries, i.e., 0.7%–27% [13,43,44,45]. This difference might be attributed to several factors, such as a difference in production systems, e.g., institutional farms [39] vs. private farms [41] vs. smallholders [14]; or the diagnostic strategy employed—a single or battery of tests applied in parallel or a serial pattern [33,46]. Thus, the parallel testing approach in this study might have influenced the results by reducing the number of false-negative reactions. A favorable climate may also be associated with a higher seroprevalence in irrigated areas as the bacteria can survive longer in humid environments as compared to the dry environments [14,47,48]. At the herd level, 2.5 times higher seroprevalence was found in the study, which is in agreement with previous studies from Pakistan [14,25,33]. Consumption of raw milk and occupational exposure, i.e., handling of infected animals or aborted materials, should be considered as the main transmission factors [49,50].

### Risk Factors Associated with Seroprevalence of Brucellosis

At the animal level, age was significantly associated with the seropositivity of the infection where adults were at higher risk (seven times) than the younger ones. Previous studies have reported a positive association with age in bovines [21] and small ruminants [51]. However, some of them could not confirm this association [39,41,52]. The higher prevalence in older animals can be attributed to the chronic nature of the disease, chances of exposure to the pathogen with an increase in age and mating with seropositive animals.

At the farm level, herd size associated significantly with the seropositivity where farms with >10 animals showed seven times higher risk. It may be due to the higher levels of exposure to infected/carrier animals at the farm. It is in line with the previous findings from the country where a higher prevalence was estimated at intensive farms [14,32,52].

History of abortion at the farm was associated significantly with the higher herd-level seropositivity, which agrees with the previous studies in Pakistan [14,41,52,53] and other countries [15,54,55]. Abortion in the last trimester is a characteristic sign of brucellosis, which may accompany other reproductive disorders, e.g., retention of fetal membranes, endometritis and infertility. Nevertheless, brucellosis may stay asymptomatic and remain undiagnosed. Abortive dams should be segregated and should not be able to transmit the disease indirectly by fomites as bacteria are shed heavily in the aborted materials and milk. It also poses a zoonotic threat to animal handlers and milk consumers [52].

Farm area was found to associate significantly with the seropositivity where <4 kanals posed a higher risk to the animals. A higher stocking density will always increase the chances of exposure, contamination and, hence, disease risk at the farm. This might be a reason why brucellosis was more prevalent on private farms as compared to the institutional farms in the country [39,41,51]. Our study found that the prevalence in female animals did not statistically differ from that of males. These results are in accordance with the findings of previous studies [21,39,41,52,56], where the sex of the animal was not associated with the occurrence of brucellosis. Similarly, a significant association of the antibody prevalence with the animal species could not be found in this study. These results are consistent with those of previous studies [52].

## 5. Conclusions

Brucellosis is a persistent problem of bovines in the country as well as in the district Gujranwala of Punjab, Pakistan. The infection pressure remains strong due to the presence of productive/mature animals, a high stocking density and a history of reproductive disorders at these farms. Such occurrence poses a direct zoonotic threat to animal handlers and the milk consumers of the area. Strict biosecurity, quarantine measures and routine screening of animals should be adopted on these farms. In case of an abortion, animal handlers should adopt protective measures and infected animals should be segregated, and the aborted material disposed-off and the premises disinfected to ensure prevention of transmission. Milk from infected animals should not be fed to other domestic animals, e.g., dogs and cats. Pasteurization of milk is recommended before consumption. Moreover, disease awareness programs would be helpful. Diagnostic laboratories need to be enabled and updated with the appropriate biosafety measures to perform isolation of the disease-causing agents in infected animals. The new diagnostic tests needed to be standardized and validated according to the disease situation of the area.

## Figures and Tables

**Table 1:** Seroprevalence of brucellosis in bovines using a parallel diagnostic approach in Gujranwala District, Pakistan.

Tehsil	Cattle				Buffalo				Overall Positive (%)
	Samples Collected	RBPT Positive (%)	ELISA Positive (%)	Total Positive (%)	Samples Collected	RBPT Positive (%)	ELISA Positive (%)	Total Positive (%)	
Gujranwala	28	5 (17.8%)	8 (28.5%)	10 (35.7%)	30	5 (16.6%)	7 (23.3%)	8 (26.6%)	18 (31.0%)
Nowshera Virkan	27	5 (18.5%)	6 (22.2%)	6 (22.2%)	27	7 (25.9%)	9 (33.3%)	9 (33.3%)	15 (27.8%)
Wazirabad	27	1 (3.7%)	2 (7.4%)	2 (7.4%)	28	4 (14.2%)	7 (25%)	7 (25%)	9 (16.4%)
Kamoke	26	2 (7.6%)	4 (15.3%)	4 (15.3%)	27	1 (3.7%)	4 (14.8%)	4 (14.8%)	8 (15.1%)
Total	108	13 (12%)	20 (18.5%)	22 (20.4%)	112	17 (15.1%)	27 (24.1%)	28 (25%)	50 (22.7%)

RBPT = Rose Bengal Plate Test, ELISA = Enzyme-Linked Immunosorbent Assay.

**Table 2:** Summary of the animal-level variables for the occurrence of brucellosis included in the initial multivariable model.

Variable	Response Categories	No. Tested (%)	No. Positive (%)
Species	Buffalo	112 (51%)	28 (25.0%)
	Cattle	108 (49%)	22 (20.4%)
Age	Adult	177 (80%)	48 (27.1%)
	Young	43 (20%)	2 (4.7%)
Sex	Female	189 (86%)	48 (25.4%)
	Male	31 (14%)	2 (6.5%)
Breeding method	Artificial insemination	78 (35%)	19 (24.4%)
	Natural mating	142 (65%)	31 (21.8%)

**Table 3:** Summary of the farm-level variables included in the initial multivariable model.

Variable	Response Categories	Responses n (%)	Odds Ratio	95% Confidence Interval	*p*-Value
Farm Related Variables					
Farm type	Traditional rural *	29 (63.0)			0.93
	Semi-commercial	17 (37.0)	1.09	0.14–8.39	
Herd size	≤10	11 (23.9)			0.027
	>10	35 (76.1)	8.1	1.25–72.35	
Presence of goat	Yes	17 (37.0)	1.63	0.27–10.71	0.592
	No	29 (63.0)			
Presence of sheep	Yes	11 (23.9)	0.44	0.05–3.13	0.412
	No	35 (76.1)			
Fencing	Yes	31 (67.4)	0.77	0.11–4.78	0.777
	No	15 (32.6)			
Neighboring farm distance	Adjacent	10 (21.7%)			0.278
	Far	36 (78.3%)	0.35	0.04–2.29	
Breeding method	Only artificial insemination	4 (8.7)			0.766
	Only natural mating	6 (13.0)	4.31	0.08–425.87	
	Both	36 (78.3)	2.9	0.11–131.2	
History of abortion at the farm	Yes	20 (43.5)	7.59	1.38–56.19	0.019
	No	26 (56.5)			
Farm area	Small < 4 Kanals *	35 (76.1)	8.42	0.94–99.25	0.056
	Large ≥ 4 Kanals	11 (23.9)			

* These types of farms have covered and uncovered areas, but without any specified proportion. The covered area consists of completely closed room/s without proper ventilation and a simple roof structure called a “chappar”. The rooms are used for protection from cold weather during the winter season, while the roof structure along with trees is used for protection during the summer and the monsoon season. * It is a local unit commonly used for measuring land, where 1 kanal = 4500 square feet.

**Table 4:** Summary of the animal-level and farm-level risk factors associated with the occurrence of brucellosis in the final model.

Variables	Response Categories	Odds Ratio	95% Confidence Interval	*p*-Value
Animal-level variables				
Age	Adult	7.63	2.22–47.93	<0.001
	Young	1		
Farm-level variables				
Herd size	>10	7.83	1.48–52.43	0.021
	≤10	1		
History of abortion at the farm	Yes	5.60	1.29–31.29	0.030
	No	1		
Farm area	Small < 4 Kanals	6.39	1.21–43.26	0.037
	Large ≥ 4 Kanals	1		

## Supplementary Materials

The following are available online at https://www.mdpi.com/article/10.3390/ani11061744/s1, Table S1: Results of Cohen’s kappa test for RBPT and I-ELISA. Table S2: Differentiated test results for cows. Table S3: Differentiated test results for buffaloes.

## References

[1] Corbel M.J. (2006). Brucellosis in Humans and Animals.

[2] Diaz-Aparicio E. (2013). Epidemiology of brucellosis in domestic animals caused by *Brucella melitensis*, *Brucella suis* and *Brucella abortus*. Rev. Sci. Tech..

[3] Buhmann G., Paul F., Herbst W., Melzer F., Wolf G., Hartmann K., Fischer A. (2019). Canine Brucellosis: Insights into the Epidemiologic Situation in Europe. Front. Vet. Sci..

[4] Alamian S., Dadar M. (2020). *Brucella melitensis* infection in dog: A critical issue in the control of brucellosis in ruminant farms. Comp. Immunol. Microbiol. Infect. Dis..

[5] Saleem M.Z., Akhtar R., Aslam A., Rashid M.I., Chaudhry Z.I., Manzoor M.A., Shah B.A., Ahmed R., Yasin M. (2019). Evidence of *Brucella abortus* in non-preferred caprine and ovine hosts by real-time PCR assay. Pak. J. Zool.

[6] Khan T.I., Ehtisham-ul-Haque S., Waheed U., Khan I., Younus M., Ali S. (2018). Milk Indirect-ELISA and Milk Ring Test for Screening of Brucellosis in Buffaloes, Goats and Bulk Tank Milk Samples Collected from Two Districts of Punjab, Pakistan. Pak. Vet. J..

[7] Álvarez J., Sáez J.L., García N., Serrat C., Pérez-Sancho M., González S., Ortega M.J., Gou J., Carbajo L., Garrido F. (2011). Management of an outbreak of brucellosis due to *B. melitensis* in dairy cattle in Spain. Res. Vet. Sci..

[8] OIE-WAHIS (2015). World Animal Health Information System 2015.

[9] Abedi A.-S., Hashempour-Baltork F., Alizadeh A.M., Beikzadeh S., Hosseini H., Bashiry M., Taslikh M., Javanmardi F., Sheidaee Z., Sarlak Z. (2020). The prevalence of *Brucella* spp. in dairy products in the Middle East region: A systematic review and meta-analysis. Acta Trop..

[10] Dadar M., Shahali Y., Whatmore A.M. (2019). Human brucellosis caused by raw dairy products: A review on the occurrence, major risk factors and prevention. Int. J. Food Microbiol..

[11] Galinska E.M., Zagórski J. (2013). Brucellosis in humans-etiology, diagnostics, clinical forms. Annal. Agric. Environ. Med..

[12] Amin K.M., Rahman M.B., Rahman M.S., Han J.C., Park J.H., Chae J.S. (2005). Prevalence of *Brucella* antibodies in sera of cows in Bangladesh. J. Vet. Sci..

[13] Silva I., Dangolla A., Kulachelvy K. (2000). Seroepidemiology of *Brucella abortus* infection in bovids in Sri Lanka. Prev. Vet. Med..

[14] Arif S., Thomson P.C., Hernandez-Jover M., McGill D.M., Warriach H.M., Hayat K., Heller J. (2019). Bovine brucellosis in Pakistan; an analysis of engagement with risk factors in smallholder farmer settings. Vet. Med. Sci..

[15] Deka R.P., Magnusson U., Grace D., Lindahl J. (2018). Bovine brucellosis: Prevalence, risk factors, economic cost and control options with particular reference to India-a review. Infec. Ecol. Epidemiol..

[16] Matope G., Bhebhe E., Muma J., Lund A., Skjerve E. (2011). Risk factors for *Brucella* spp. infection in smallholder household herds. Epidemiol. Infect..

[17] Rehman A., Jingdong L., Chandio A.A., Hussain I. (2017). Livestock production and population census in Pakistan: Determining their relationship with agricultural GDP using econometric analysis. Inf. Process. Agric..

[18] Afzal M. (2010). Re-designing smallholder dairy production in Pakistan. Pak. Vet. J..

[19] Rehman A., Nijhof A.M., Sauter-Louis C., Schauer B., Staubach C., Conraths F.J. (2017). Distribution of ticks infesting ruminants and risk factors associated with high tick prevalence in livestock farms in the semi-arid and arid agro-ecological zones of Pakistan. Parasites Vectors.

[20] Abubakar M., Javed Arshed M., Hussain M., Ehtisham ul H., Ali Q. (2010). Serological evidence of *Brucella abortus* prevalence in Punjab province, Pakistan--a cross-sectional study. Transbound. Emerg. Dis..

[21] Gul S.T., Khan A., Rizvi F., Hussain I. (2014). Sero-prevalence of brucellosis in food animals in the Punjab, Pakistan. Pak. Vet. J..

[22] Shehzad A., Rantam F.A., Masud A., Ahmed S. (2020). Seroprevalence and potential risk factors associated with brucellosis in the Desert Thal of Pakistan. Human Vet. Med..

[23] (2020). Economic Survey of Pakistan 2019–2020 Chapter 2: Agriculture.

[24] Punjab L.C. (2018). First Real Time (Door to Door) Livestock Census.

[25] Nasir A., Parveen Z., Shah M., Rashid M. (2004). Seroprevalence of brucellosis in animals at government and private livestock farms in Punjab. Pak. Vet. J..

[26] Neha A., Kumar A., Ahmed I. (2017). Comparative efficacy of serological diagnostic methods and evaluation of polymerase chain reaction for diagnosis of bovine brucellosis. Iran. J. Vet. Res..

[27] OIE (2009). Terrestrial Manual; 2.4.3; Bovine brucellosis. https://www.id-vet.com/produit/id-screen-brucellosis-serum-indirect-multi-species/.

[28] R Core Team (2021). R: A Language and Environment for Statistical Computing.

[29] RStudio Team (2021). RStudio: Integrated Development for R.

[30] Gisev N., Bell J.S., Chen T.F. (2013). Interrater agreement and interrater reliability: Key concepts, approaches, and applications. Res. Soc. Adm. Pharm..

[31] McHugh M.L. (2012). Interrater reliability: The kappa statistic. Biochem. Med..

[32] Khan S.I., Muti-ur-Rehmana S.M., Khanc A., Khand A., Shakeeld M., Shah M.A., Naseerd Z. (2017). Patho-Epidemiology of Bovine Brucellosis in Aza-kheli Buffalo in Pakistan. Veterinaria.

[33] Ali S., Akhter S., Neubauer H., Melzer F., Khan I., Abatih E.N., El-Adawy H., Irfan M., Muhammad A., Akbar M.W. (2017). Seroprevalence and risk factors associated with bovine brucellosis in the Potohar Plateau, Pakistan. BMC Res. Notes.

[34] Khurana S.K., Sehrawat A., Tiwari R., Prasad M., Gulati B., Shabbir M.Z., Chhabra R., Karthik K., Patel S.K., Pathak M. (2021). Bovine brucellosis—A comprehensive review. Vet. Q..

[35] Abubakar M., Mansoor M., Arshed M.J. (2012). Bovine Brucellosis: Old and New Concepts with Pakistan Perspective. Pak. Vet. J..

[36] OIE (2018). Brucellosis (*Brucella abortus*, *B. melitensis* and *B. suis*) (Infection with *B. abortus*, *B. melitensis* and *B. suis*). OIE Terristrial Manual.

[37] Gusi A.M., Bertu W.J., de Miguel M.J., Dieste-Pérez L., Smits H.L., Ocholi R.A., Blasco J.M., Moriyón I., Muñoz P.M. (2019). Comparative performance of lateral flow immunochromatography, iELISA and Rose Bengal tests for the diagnosis of cattle, sheep, goat and swine brucellosis. PLoS Neglec. Trop. Dis..

[38] Ducrotoy M.J., Muñoz P.M., Conde-Álvarez R., Blasco J.M., Moriyón I. (2018). A systematic review of current immunological tests for the diagnosis of cattle brucellosis. Prev. Vet. Med..

[39] Jamil T., Melzer F., Saqib M., Shahzad A., Khan Kasi K., Hammad Hussain M., Rashid I., Tahir U., Khan I., Haleem Tayyab M. (2020). Serological and Molecular Detection of Bovine Brucellosis at Institutional Livestock Farms in Punjab, Pakistan. Int. J. Environ. Res. Public Health.

[40] Ghodasara S., Roy A., Bhanderi B. (2010). Comparison of Rose Bengal plate agglutination, standard tube agglutination and indirect ELISA tests for detection of *Brucella* antibodies in cows and buffaloes. Vet. World.

[41] Ullah Q., Jamil H., Lodhi L.A., Qureshi Z.I., Ullah S., Jamil T., Khan I., Bashir S., Wazir I., Sallam M.A. (2019). Brucellosis is significantly associated with reproductive disorders in dairy cattle of Punjab, Pakistan. Pak. J. Zool..

[42] Saeed U., Ali S., Latif T., Rizwan M., Saif A., Iftikhar A., Ghulam Mohayud Din Hashmi S., Khan A.U., Khan I., Melzer F. (2020). Prevalence and Spatial Distribution of Animal Brucellosis in Central Punjab, Pakistan. Int. J. Environ. Res. Public Health.

[43] Ahasan M.S., Rahman M.S., Rahman A.A., Berkvens D. (2017). Bovine and Caprine Brucellosis in Bangladesh: Bayesian evaluation of four serological tests, true prevalence, and associated risk factors in household animals. Trop. Anim. Health Prod..

[44] Mombeni E.G., Mombeni M.G., Khalaj M., Asadi R., Rezaei A.A., Amiri K., Bromand S., Kenarkohi M., Mombeni A.G. (2014). Seroprevalence of brucellosis in livestock in Khuzestan province, Southwest of Iran, 2008–2012. İstanbul Üniversitesi Vet. Fakül. Derg..

[45] Zadon S., Sharma N. (2015). Seroprevalence of bovine brucellosis in different agro-climatic regions of Punjab. Asian J. Anim. Vet. Adv..

[46] Gul S.T., Khan A., Ahmad M., Rizvi F., Shahzad A., Hussain I. (2015). Epidemiology of brucellosis at different livestock farms in the Punjab, Pakistan. Pak. Vet. J..

[47] Ahmed R., Muhammad K., Rabbani M., Khan M.S. (2017). Spatial distribution of soil borne brucella species specific DNA in Punjab, Pakistan. Pak. J. Zool..

[48] Rodríguez-Morales A.J. (2013). Climate change, climate variability and brucellosis. Recent Pat. Anti Infec. Drug Dis..

[49] Hakeem M., Saeed S. (2019). Brucellosis: A case report and literature review. J. Postgrad. Med. Ed. Res..

[50] Mukhtar F. (2010). Brucellosis in a high risk occupational group: Seroprevalence and analysis of risk factors. J. Pak. Med. Assoc..

[51] Ullah Q., Jamil T., Melzer F., Saqib M., Hussain M.H., Aslam M.A., Jamil H., Iqbal M.A., Tahir U., Ullah S. (2020). Epidemiology and Associated Risk Factors for Brucellosis in Small Ruminants Kept at Institutional Livestock Farms in Punjab, Pakistan. Front. Vet. Sci..

[52] Saeed U., Ali S., Khan T.M., El-Adawy H., Melzer F., Khan A.U., Iftikhar A., Neubauer H. (2019). Seroepidemiology and the molecular detection of animal brucellosis in Punjab, Pakistan. Microorganisms.

[53] Ismail M., Ahmad I., Khan M.S., Ullah S., Malik M.I., Muhammad K., Safder K., Jelani G., Baber A., Jan A.A. (2018). Seroprevalance of *Brucella* abortus in cattle and buffaloes in district Rajanpur, Punjab, Pakistan. Pure Appl. Biol..

[54] Boukary A.R., Saegerman C., Abatih E., Fretin D., Alambédji Bada R., De Deken R., Harouna H.A., Yenikoye A., Thys E. (2013). Seroprevalence and potential risk factors for *Brucella* spp. infection in traditional cattle, sheep and goats reared in urban, periurban and rural areas of Niger. PLoS ONE.

[55] Lindahl E., Sattorov N., Boqvist S., Sattori I., Magnusson U. (2014). Seropositivity and risk factors for *Brucella* in dairy cows in urban and peri-urban small-scale farming in Tajikistan. Trop. Anim. Health Prod..

[56] Munir R., Farooq U., Fatima Z., Afzal M., Anwar Z., Jahangir M. (2011). Sero-prevalence of brucellosis in bovines at farms under different management conditions. Br. J. Dairy Sci..

