# Supplementary material for: Seroprevalence and Associated Risk Factors of Bovine Brucellosis in District Gujranwala, Punjab, Pakistan
Source: Animals (Basel). 2021 Jun 11;11(6):1744. doi: 10.3390/ani11061744 (PMC8230616; doi:10.3390/ani11061744)
Supplement: Supplementary file 1 [file animals-11-01744-s001.zip › animals-1193515-supplementary.pdf]

Supplementary Table S1: Results of Cohen's kappa test for RBPT and I-ELISA

Test used	ELISA +ve	ELISA -ve
**RBPT +ve**	27	03
**RBPT -ve**	20	170

RBPT = Rose Bengal Plate Test; ELISA = Enzyme-Linked Immunosorbent Assay

Percent agreement = 89.54; Cohen's kappa coefficient = 0.64, Standard Error (SE) = 0.067, 95% CI = 0.510 – 0.773

Supplementary Table S2: Differentiated test results for cows

Test used	ELISA +ve	ELISA -ve
**RBPT +ve**	11	02
**RBPT -ve**	09	86

RBPT = Rose Bengal Plate Test; ELISA = Enzyme-Linked Immunosorbent Assay

Supplementary Table S3: Differentiated test results for buffaloes

Test used	ELISA +ve	ELISA -ve
**RBPT +ve**	16	01
**RBPT -ve**	11	84

RBPT = Rose Bengal Plate Test; ELISA = Enzyme-Linked Immunosorbent Assay
